# Mre11 exonuclease activity removes the chain-terminating nucleoside analog gemcitabine from the nascent strand during DNA replication

**DOI:** 10.1126/sciadv.aaz4126

**Published:** 2020-05-29

**Authors:** L. Boeckemeier, R. Kraehenbuehl, A. Keszthelyi, M. U. Gasasira, E. G. Vernon, R. Beardmore, C. B. Vågbø, D. Chaplin, S. Gollins, H. E. Krokan, S. A. E. Lambert, B. Paizs, E. Hartsuiker

**Affiliations:** 1 North West Cancer Research Institute, School of Medical Sciences, Bangor University, Bangor, Gwynedd LL57 2UW, UK.; 2 Centre for Environmental Biotechnology, School of Natural Sciences, Bangor University, Bangor, Gwynedd LL57 2UW, UK.; 3 Department of Clinical and Molecular Medicine, Norwegian University of Science and Technology, NO-7491 Trondheim, Norway.; 4 Institut Curie, Paris-Saclay University, UMR3348, F-91450 Orsay, France.

## Abstract

The Mre11 nuclease is involved in early responses to DNA damage, often mediated by its role in DNA end processing. *MRE11* mutations and aberrant expression are associated with carcinogenesis and cancer treatment outcomes. While, in recent years, progress has been made in understanding the role of Mre11 nuclease activities in DNA double-strand break repair, their role during replication has remained elusive. The nucleoside analog gemcitabine, widely used in cancer therapy, acts as a replication chain terminator; for a cell to survive treatment, gemcitabine needs to be removed from replicating DNA. Activities responsible for this removal have, so far, not been identified. We show that Mre11 3′ to 5′ exonuclease activity removes gemcitabine from nascent DNA during replication. This contributes to replication progression and gemcitabine resistance. We thus uncovered a replication-supporting role for Mre11 exonuclease activity, which is distinct from its previously reported detrimental role in uncontrolled resection in recombination-deficient cells.

## Teaser

Mre11 exonuclease activity removes gemcitabine from the nascent strand during DNA replication.

## INTRODUCTION

Error-free DNA replication is essential for maintaining genome stability and for preventing the accumulation of mutations, which drive carcinogenesis. Replication fork blockage interferes with cell division and contributes to genome instability. Different sources of replication stress can impede fork progression, e.g., a lack of deoxynucleotide triphosphates (dNTPs), or obstacles (e.g., DNA lesions) in the template strand. Various mechanisms have evolved to deal with these sources of replication stress (*1*).

Homologous recombination (HR) is a well-studied mechanism involved in repair of DNA double-strand breaks (DSBs). In HR, the DNA is repaired through invasion of a broken DNA strand into a homologous DNA duplex followed by copy synthesis. Before strand invasion takes place, 5′ DSB ends are resected to create 3′ single-strand overhangs (*2*). One of the proteins responsible for end resection is Mre11, part of the Mre11/Rad50/Nbs1 (MRN) complex.

MRN is highly conserved among eukaryotes and is, together with CtIP, involved in a wide range of early responses to DNA damage, often mediated by its role in DNA end processing (*3*). Central to DNA end processing are Mre11 single-strand endonuclease and 3′ to 5′ exonuclease activities. The role of Mre11 exonuclease activity in HR has long been enigmatic, as the 3′ to 5′ polarity is in the opposite direction of the 5′ to 3′ resection required for HR. More recently, several studies suggest that the Mre11 endonuclease activity creates single-strand nicks, which serve as entry points for the Mre11 3′ to 5′ exonuclease activity directed toward the break, while other (nuclease) activities are responsible for resection away from the break (*4*–*6*). After resection, invasion of the 3′ end of the DSB into homologous DNA is mediated by Rad51, aided in humans by BRCA1, BRCA2, and several RAD51 paralogues. The invading 3′ end acts as a primer for copy synthesis (*2*).

Several Mre11 functions are independent of its nuclease activities. *Saccharomyces cerevisiae mre11* nuclease-deficient mutants are only partially sensitive to ionizing radiation and are proficient for several other phenotypes observed in *mre11* null mutants (*7*). In *Schizosaccharomyces pombe*, resection of the C-rich strand at telomere ends requires Mre11 but is independent of its nuclease activities (*8*), while an *mre11* nuclease mutant is defective in Rec12^Spo11^ removal and shows sensitivity to topoisomerase poisons but not to methyl methanesulfonate and ionizing radiation (*9*).

MRN/CtIP and other proteins involved in HR have also been implicated in the restart of stalled replication forks after they encounter an obstacle in the template strand (*10*–*16*). The precise role of the MRN complex, and especially the role of the Mre11 nuclease activity in replication fork restart, is not understood. *S. cerevisiae* Mre11 is recruited to paused replication forks and stabilizes their association with replisome components, but this function does not depend on the Mre11 nuclease activity (*17*). In *S. pombe*, enrichment of the HR protein Rad52 at stalled forks is dependent on the MRN complex (*14*) but is independent of Mre11 nuclease activity (*18*). In absence of some HR proteins in human cells (e.g., when RAD51 or BRCA2 are mutated/depleted), MRE11 exonuclease activity is responsible for uncontrolled deleterious degradation of stalled and reversed forks (*19*). However, it remains unknown whether and how MRE11 nuclease activity supports replication progression in recombination-proficient cells.

While DNA damage response mechanisms that deal with fork stalling caused by nucleotide pool depletion or obstacles on the template strand have been widely studied (*20*), less is known about mechanisms that deal with the replication blockage of the nascent strand, e.g., caused by incorporation of chain-terminating nucleotides. Nucleoside analogs are frequently used in cancer therapy. They interfere with DNA replication by inhibiting nucleotide metabolism and can act as DNA replication chain terminators after incorporation into nascent DNA (*21*). The nucleoside analog gemcitabine [2′,2′-difluoro 2′-deoxycytidine (dFdC)], used to treat a range of cancers (*22*), is a deoxycytidine analog that contains two fluorine atoms at the 2′ carbon of the sugar ring. It is a prodrug that, once transported into a cell, is phosphorylated into dFdC monophosphate (dFdCMP) by deoxycytidine kinase (dCK) and subsequently to dFdC diphosphate (dFdCDP) and dFdC triphosphate (dFdCTP) (*22*). Competing with deoxycytidine triphosphate, dFdCTP is incorporated into the nascent DNA strand during replication, which leads to chain termination after incorporation of an additional dNTP (*23*). In addition, dFdCDP acts as an inhibitor of ribonucleotide reductase (RNR), depleting the dNTP pools, thus increasing the likelihood of dFdCTP integration (*22*).

Little is known about DNA repair pathways resisting treatment with dFdC. Chinese hamster ovary (CHO) cell nucleotide excision repair (*xpd* and *ercc1*), nonhomologous end joining (*DNA-pkcs*), base excision repair (*xrcc1*), and HR (*xrcc3*) mutants are not sensitive to dFdC (*24*). In contrast, mutation of BRCA2 in CHO cells and small interfering RNA (siRNA) knockdown of human RAD51 increase dFdC resistance (*25*).

For a cell to be able to resist treatment with dFdC, the chain-terminating nucleoside analog must be removed to allow replication restart. Little is known about mechanisms that remove replication-terminating dFdC (or other chain-terminating nucleoside analogs) from nascent DNA. In vitro, the proofreading exonuclease activity of DNA polymerase ε is able to remove the chain-terminating nucleoside analog cytarabine from DNA ends (*23*), and this activity contributes to cellular cytarabine tolerance (*26*). However, this activity is not able to efficiently remove dFdCMP from a 3′ DNA end or from the penultimate (masked by another nucleotide) position (*23*). Similarly, the human-base excision repair nuclease Ape1 was shown to be able to remove l-configuration nucleoside analogs (e.g., troxacitabine) from DNA but had little activity against dFdC or other d-configuration nucleoside analogs (*27*). TDP1 is able to remove cytarabine from 3′ DNA ends in vitro, and *TDP1^−/−^* DT40 cells and mouse embryonic fibroblasts are hypersensitive to this drug, while they are not sensitive to dFdC (*28*). Hence, the identity of the enzyme(s) involved in dFdC removal has remained elusive.

We have previously shown that the nuclease activity of fission yeast Mre11 is involved in removing covalently bound Spo11 (*29*), topoisomerase I, and topoisomerase II (*9*) from DNA. After dFdC treatment, MRN proteins form nuclear foci at stalled forks in the absence of detectable DNA breaks, while siRNA knockdown of Mre11, Rad50 (*30*), and mirin treatment (*31*) causes a slight dFdC sensitivity in human cells. A recent study (*32*) in DT40 cells showed that some other nucleoside analogs (dFdC was not tested) retard replication in Mre11 nuclease mutants. However, removal of these nucleoside analogs from genomic DNA was not assessed, and it has remained unknown whether the observed phenotypes in these mutants were due to a role of Mre11 in resisting nucleotide pool imbalances (caused by treatment with nucleoside analogs) or whether Mre11 nuclease activity is involved in removal of chain-terminating nucleoside analogs. In addition, as the *MRE11* mutants used for this study were defective for both endo- and exonuclease activity, it has remained unclear whether replication delay was caused by an endo- or exonuclease defect. We thus decided to test the potential roles for the Mre11 endo- and exonuclease activities in removing the chain-terminating nucleoside analog dFdC from genomic DNA during DNA replication in several model organisms.

## RESULTS

### Creation of a nucleotide salvage pathway in *S. pombe*

**Fig. 1:**
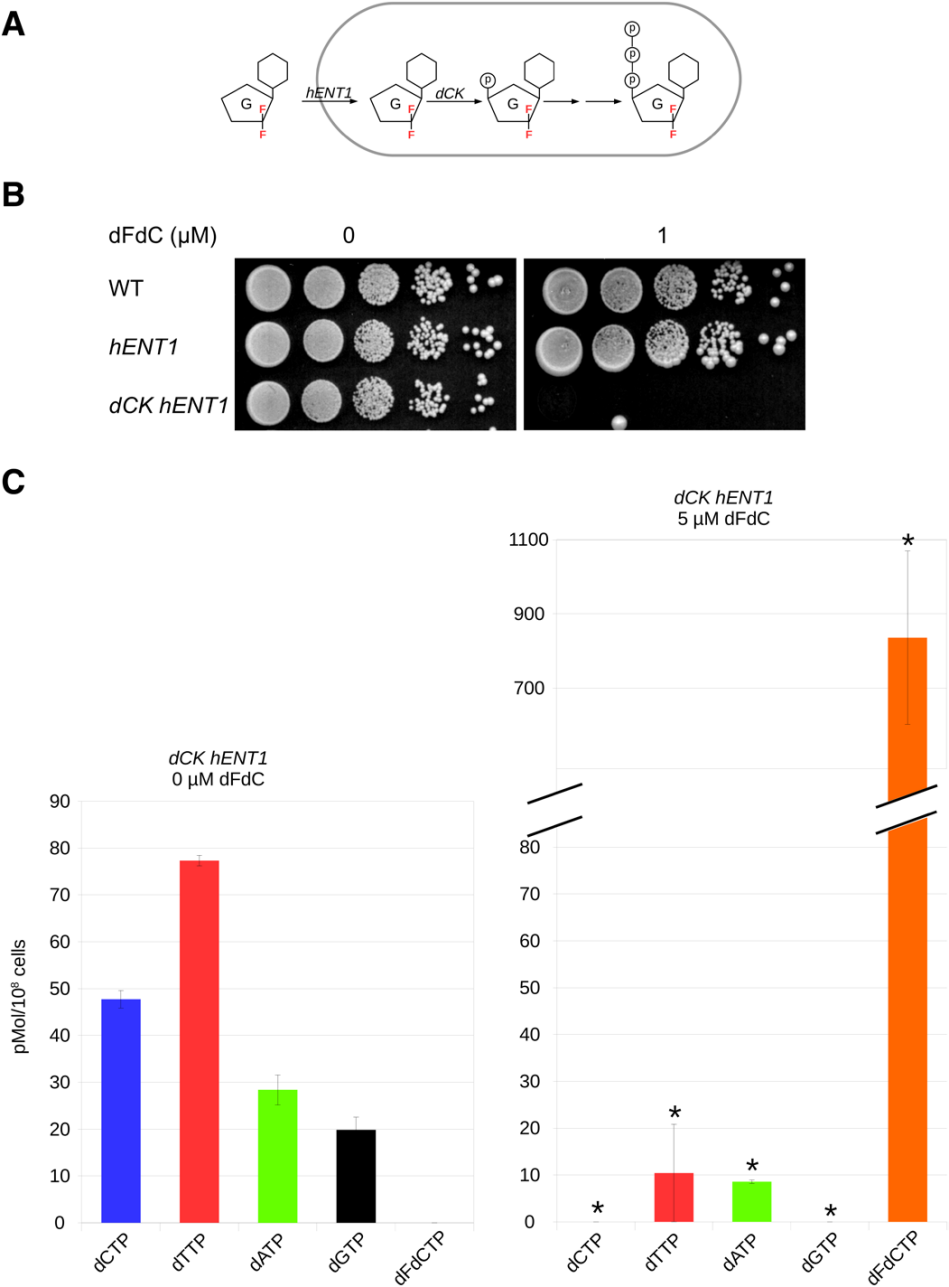
Creation of a nucleotide salvage pathway in *S. pombe*. (**A**) Integration of the genes encoding for the *hENT1* and the *dCK* into the *S. pombe* genome. This facilitates the import of dFdC into *S. pombe* cells and the subsequent phosphorylation of dFdC into dFdCMP and through two subsequent phosphorylation steps into dFdCTP. (**B**) Wild-type (WT) cells without *hENT1* and *dCK*, as well as cells expressing only *hENT1*, are not sensitive to 1 μM dFdC; cells expressing both *hENT1* and *dCK* are sensitive. (**C**) Treating *hENT1 dCK* strains with dFdC led to a reduction in dNTP levels, and the appearance of dFdCTP, resulting from the triple phosphorylation of dFdC. This shows that dFdC is transformed into dFdCTP in cells containing *hENT1* and *dCK*. Asterisk indicates a statistically significant difference with untreated cells (*t* test, *P* < 0.05; *n* = 3). G, gemcitabine; F, fluorine; P, Phosphate.

As *S. pombe* does not have a functional nucleotide salvage pathway that would allow the uptake and phosphorylation of dFdC, we inserted the genes encoding for the human equilibrative nucleoside transporter 1 (*hENT1*) (*33*) and the human deoxycytidine kinase (*dCK*) (*34*), both under the control of the constitutive *adh* promoter, into the *S. pombe* genome. This facilitates the import of dFdC into *S. pombe* cells and the subsequent phosphorylation of dFdC into dFdCMP and, subsequently, through two phosphorylation steps by endogenous nucleoside monophosphate and nucleoside diphosphate kinases (*35*) into dFdCTP (see Fig. 1A).

As shown in Fig. 1B, strains without *hENT1* and *dCK*, as well as cells with only *hENT1* but not *dCK*, were resistant to 1 μM dFdC, while cells expressing both hENT1 and dCK were sensitive. We measured intracellular dNTP and dFdCTP levels, as shown in Fig. 1C, treating *hENT1 dCK* strains with dFdC led to the appearance of dFdCTP, resulting from the triple phosphorylation of dFdC. Similar to the effects observed in human cells (*22*), *S. pombe* dNTP pools were reduced after dFdC treatment in hENT1 dCK cells. We could detect dFdCMP, dFdCDP, and dFdCTP in *hENT1 dCK* cells treated with dFdC but not in dFdC-treated cells, which do not contain *hENT1* and *dCK* (fig. S1). These results show that insertion of the *hENT1* transporter and *dCK* kinase successfully creates a nucleotide salvage pathway in *S. pombe*, which sensitizes cells to dFdC.

### MRN and Ctp1 promote resistance to dFdC and removal from genomic DNA

**Fig. 2:**
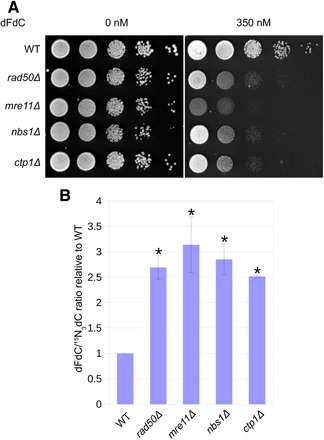
MRN and Ctp1 promote resistance to dFdC and removal from genomic DNA. (**A**) *mrn*/*ctp1* deletion mutants are sensitive to dFdC. Representative example of three experiments. (**B**) The dFdC/hdC ratio is significantly increased in *mrn*/*ctp1* deletion mutants compared to WT. Error bars depict SE; asterisk indicates statistically significant difference with WT (*t* test, *P* < 0.05; *n* = 3).

To study whether the MRN complex and the *S. pombe* Sae2/CtIP homolog Ctp1 (*36*) contribute to dFdC resistance, we tested the sensitivity of deletion mutants to dFdC and found that these were all sensitive (Fig. 2A). Next, to assess whether these mutants were deficient in removing dFdC from genomic DNA after incorporation, we quantified dFdC in genomic DNA. Briefly (for details, see Materials and Methods), wild-type (WT) and mutant cells were treated for 3 hours with dFdC and a stable “heavy” isotope of deoxycytidine, ^15^N_3_dC (hdC). Genomic DNA was isolated and subsequently enzymatically digested and dephosphorylated into single nucleosides. Using liquid chromatography–tandem mass spectrometry (LC-MS/MS), the amounts of dFdC and hdC in genomic DNA were quantified. To correct for differences in replication progression between the strains, we calculated the dFdC/hdC ratio, which reflects the amount of dFdC in genomic DNA corrected for the total amount of replication taken place during dFdC/hdC treatment. As shown in Fig. 2B, the dFdC/hdC ratios were significantly increased ~2.5- to 3-fold in the deletion mutants compared to WT.

Several studies have shown that dFdC is incorporated into genomic DNA during S phase and that this is responsible for its cytotoxic effect, rather than dNTP pool depletion (*23*, *37*, *38*). In our study, under the treatment conditions used to measure the dFdC/hdC ratio (50 μM dFdC), cells are arrested in early S phase (as shown in Fig. 4; cells are arrested when exposed to 2 μM dFdC). As dFdC is not incorporated outside of S phase and cells do not progress through S phase at high dFdC concentrations, the increase in the dFdC/hdC ratio in the deletion mutants is best explained with a deficiency in removing dFdC from the nascent strand during DNA replication, suggesting that dFdC removal is promoted by the MRN complex and Ctp1.

### Mre11 nuclease activity contributes to dFdC resistance and removal

**Fig. 3:**
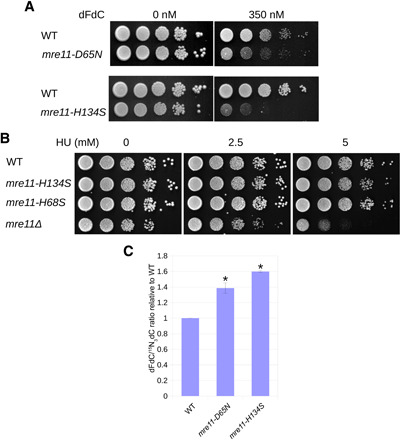
Mre11 nuclease activity contributes to dFdC resistance and removal. (**A**) The *mre11* nuclease mutants *mre11-D65N* and *mre11-H134S* are sensitive to dFdC. Representative example of three experiments. (**B**) *mre11* nuclease mutants are not sensitive against hydroxyurea (HU) concentrations, which sensitize an *mre11* deletion mutant. (**C**) The dFdC/hdC ratio is significantly increased in *mre11* nuclease mutants compared to WT. Error bars depict SE; asterisk indicates statistically significant difference with WT (*t* test, *P* < 0.05; *n* = 3 for WT and *mre11-H134S*; *n* = 6 for *mre11-D65N*).

To assess a potential contribution of Mre11 nuclease activity to dFdC resistance, we tested the sensitivity of two different *mre11* nuclease mutants (impaired for both endo- and exonuclease activity), *mre11-D65N* (*9*, *39*) and *mre11-H134S* (*40*), to dFdC. As shown in Fig. 3A, both nuclease mutants are sensitive to dFdC.

dFdC acts as a chain terminator but also as an inhibitor of RNR, thus decreasing dNTP pools. To test whether the sensitivity of *S. pombe mre11* nuclease dead mutants can be (partially) attributed to a potential role in resisting dNTP depletion, we tested *mre11-H134S* and the exonuclease mutant *mre11-H68S* (see also below) (*40*) for sensitivity against hydroxyurea (HU), another RNR inhibitor. As shown in Fig. 3B, these *mre11* mutants were not hypersensitive to HU at concentrations that substantially sensitize *mre11*Δ cells. While this confirms that the MRN complex is involved in resisting dNTP pool depletion (*41*), it suggests that this function is not mediated by *mre11* nuclease activity and that the sensitivity of the *mre11* nuclease mutants to dFdC is not due to a role of this activity in resisting dNTP depletion but is more likely explained by its dFdC removal defect. We measured the dFdC/hdC ratios and found that these are increased in both *mre11-D65N* and *mre11-H134S* mutants (Fig. 3C), suggesting that the Mre11 nuclease activity removes dFdC from genomic DNA during replication.

The increase in genomic dFdC/hdC ratio in *mre11* nuclease mutants compared to WT shows that the Mre11 nuclease activity preferentially removes replication-blocking dFdC over hdC (which is chemically indistinguishable from dC). We also found that an *S. pombe* deletion of *exo1*, encoding a nuclease that has been implicated in long-range resection at the replication fork (*42*), does not show an increase in the dFdC/hdC ratio (see fig. S2). This suggests that indiscriminate (having no preference for dFdC, hdC, or natural deoxynucleotides) long-range resection of stalled forks does not affect the dFdC/hdC ratio in our assay. These observations indicate that the increased dFdC/hdC ratio in the *mre11* nuclease mutants is not due to a role of Mre11 in uncontrolled resection of nascent DNA strands at the replication fork.

While the dFdC/hdC ratio in *mre11*Δ shows a ~3-fold increase compared to WT (Fig. 2B), the increase in the two *mre11* nuclease mutants is reduced to ~1.5-fold. This might reflect that some dFdC is removed by an alternative (MRN-dependent) activity or that the *mre11* nuclease mutants are only partially nuclease deficient.

### Mre11 nuclease activity supports replication progression in the presence of dFdC

Data presented so far suggest that Mre11 nuclease activity contributes to dFdC resistance by removing chain-terminating dFdC from nascent DNA, thus supporting replication progression. We therefore assessed replication progression using flow cytometry in synchronized WT and *mre11-D65N* strains. Temperature sensitive *nda3-KM311* cells were arrested at G_2_-M and, subsequently, released after which they performed synchronous mitosis followed by replication (*43*).

**Fig. 4:**
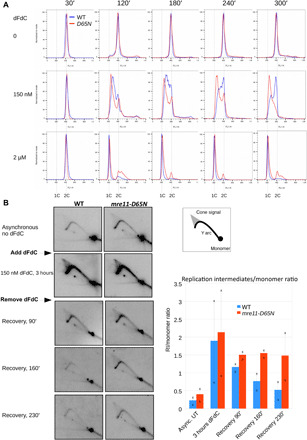
Mre11 nuclease activity supports replication progression in the presence of dFdC. (**A**) Flow cytometric analysis of WT (blue) and *mre11-D65N* (red) cells, synchronized and released in G_2_ in the presence of 0, 150 nM, and 2 μM dFdC. This is an alternative presentation of data shown in fig. S3, allowing direct comparison between WT and *mre11-D65N* cells. Flow cytometric profiles in the absence of dFdC and in the presence of 2 μM dFdC show little difference between WT and *mre11-D65N* cells. In the presence of 150 nM dFdC, WT cells (blue) slowly progress through S phase from 1C to 2C, whereas the great majority of *mre11-D65N* cells (red) fail to progress and display a 1C content throughout the time course. Representative example of three experiments. (**B**) Quantification of the combined replication intermediates (Y arc and cone structures) relative to the (nonreplicating) monomer spot shows that replication intermediates persist in the *mre11-D65N* mutant, suggesting that Mre11 nuclease activity is required for recovery from dFdC treatment. Left: Representative example of two experiments. Right: Quantification of replication intermediate signal/monomer spot ratio, relative to the ratio in untreated asynchronous cells. Average of two experiments, “x” depicts the values from individual experiments from which the average was calculated. The replication intermediates (RI)/monomer ratio is significantly higher in *mre11-D65N* compared to WT (pairwise comparison using paired *t* test, *P* = 0.034).

As shown in fig. S3A, after release from the G_2_ block, the shift to G_1_ (due to mitosis) was barely visible in untreated WT and *mre11-D65N* cells. This is because *S. pombe* cells have a very short G_1_ phase and will initiate DNA replication directly after mitosis, before cytokinesis (see fig. S3B for schematic). When WT and *mre11-D65N* cells were treated with 2 μM dFdC, replication was inhibited and cells were blocked in early S phase. As shown in Fig. 4A (a different representation of the data shown in fig. S3A), in both untreated cells and cells treated with 2 μM dFdC, there was little difference in replication progression between WT and *mre11-D65N* cells. However, in cells treated with 150 nM dFdC, we observed delayed S phase progression in WT and pronounced replication arrest in *mre11-D65N* cells.

We also compared the replication fork progression between WT and *mre11-D65N* using two-dimensional (2D) gel electrophoresis, which allows the analysis of replication intermediates (*44*). Asynchronous WT and mutant cells were treated with 150 nM dFdC for 3 hours, after which dFdC was washed out to allow recovery. As shown in Fig. 4B, replication intermediates (including Y arc and cone signal) accumulated in both strains upon dFdC treatment, consistent with a global slowdown in S phase progression. During recovery, DNA replication intermediates persisted longer in *mre11-D65N* compared to WT, indicating that replication fork progression was delayed in the absence of Mre11 nuclease activity. Overall, the replication intermediates/monomer ratio is significantly higher in *mre11-D65N* compared to WT (*P* = 0.034, pairwise comparison using paired *t* test). These results show that Mre11 nuclease activity supports replication progression after dFdC treatment.

### The *S. pombe mre11-H68S* mutant is mildly sensitive to dFdC

**Fig. 5:**
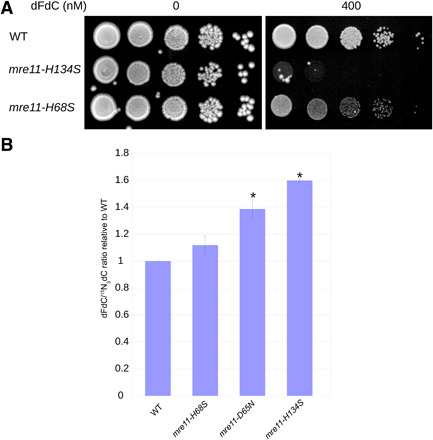
The *S. pombe mre11-H68S* mutant is mildly sensitive to dFdC. (**A**) While the *mre11-H134S* nuclease mutant, which is impaired for both endo- and exonuclease activity, is sensitive to 400 nM dFdC, the *mre11-H68S* mutant is only mildly sensitive. Representative example of three experiments. (**B**) The dFdC/hdC ratio is significantly increased in *mre11-D65N* and *mre11H134S* (same data as shown in Fig. 3C to allow direct comparison) compared to WT, *mre11-H68S* shows a small but insignificant increase. Error bars depict SE. Asterisk indicates a statistically significant difference with WT (*t* test, *P* < 0.05; *n* = 3 for WT and *mre11-H134S*, *n* = 6 for *mre11-D65N*, and *n* = 3 for *mre11-H68S*).

Mre11 has both single-strand endonuclease and 3′ to 5′ exonuclease activities. As the polarity of the 3′ to 5′ exonuclease activity is ideally suited to remove replication blocking nucleoside analogs from the growing 3′ end of the nascent DNA strand, we tested whether an *S. pombe mre11-H68S* mutant is sensitive to dFdC. This mutant was previously assumed to be defective for the 3′ to 5′ exonuclease activity based on the absence of exonuclease activity in an equivalent *Pyrococcus furiosus mre11* mutant in vitro (*40*). As shown in Fig. 5A, the *mre11-H68S* mutant was mildly sensitive to dFdC compared to WT but less sensitive than the *mre11-H134S* mutant, which is deficient for both endo- and exonuclease activities. We also determined the dFdC/hdC ratio in this mutant and found that it showed a small insignificant increase compared to WT (see Fig. 5B). However, it was previously shown that the equivalent *S. cerevisiae* mutant protein Mre11-H59S showed reduced, but not abolished, 3′ to 5′ exonuclease activity in biochemical assays (*4*). Thus, while the slight sensitivity of the *mre11-H68S* mutant suggests that the exonuclease activity contributes to dFdC resistance, the extent of this role might be masked by residual exonuclease activity in the *mre11-H68S* mutant.

### Mre11 3′ to 5′ exonuclease activity contributes to dFdC resistance and removal in DT40 and MRC-5 cells

**Fig. 6:**
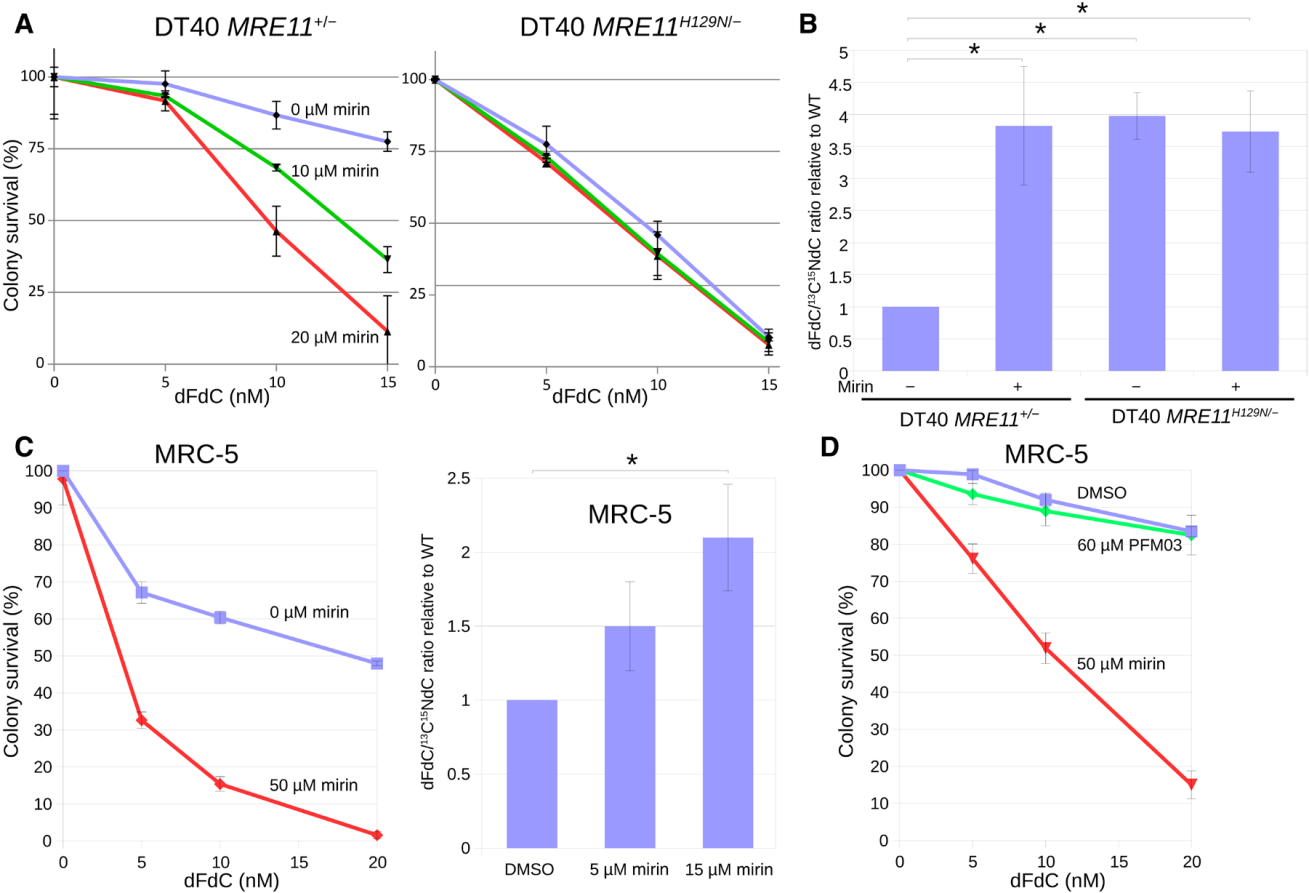
Mre11 3′ to 5′ exonuclease activity contributes to dFdC resistance and removal in DT40 and MRC-5 cells. (**A**) *MRE11^H129N/−^* DT40 cells are sensitive to dFdC compared to *MRE11^+/−^* cells in the absence of the exonuclease inhibitor mirin (blue line). In *MRE11^+/−^* cells, the addition of mirin (green and red lines) increases dFdC sensitivity, whereas in *MRE11^H129N/−^* cells, mirin does not sensitize against dFdC beyond the sensitivity caused by the nuclease mutation. Error bars depict SE; *n* = 3. (**B**) Mirin treatment increases the dFdC/hdC ratio in *MRE11^+/−^* cells. *MRE11^H129N/−^* cells also show an increased dFdC/hdC ratio, but the addition of mirin to the nuclease mutant cells does not further increase the dFdC/hdC ratio. Error bars depict SE. Asterisk indicates statistically significant difference with *MRE11^+/−^* cells (*t* test, *P* < 0.05; *n* = 7). (**C**) Mirin treatment increases dFdC sensitivity (left; error bars depict SE; *n* = 3) and increases the dFdC/hdC ratio in MRC-5 cells (right; error bars depict SE; asterisk indicates statistical significance; *t* test, *P* < 0.05; *n* = 7). (**D**) The Mre11 endonuclease inhibitor PFM03 does not sensitize MRC-5 cells to dFdC. Error bars depict SE; *n* = 3. DMSO, dimethyl sulfoxide.

As results obtained using the *S. pombe mre11-H68S* mutant were not conclusive and to determine whether the role of the Mre11 nuclease activity has been conserved in higher eukaryotes, we decided to study dFdC resistance and removal in chicken DT40 and human MRC-5 cells. First, to assess the contribution of Mre11 nuclease activity to dFdC removal and resistance in vertebrate cells, we created an *MRE11^H129N/−^* nuclease mutant (defective for both endo- and exonuclease activity) in DT40 cells, expressing the mutant protein from its endogenous promotor, preserving the intron/exon structure of the gene (see Materials and Methods for details). As shown in Fig. 6A (blue lines), this mutant was sensitive to dFdC compared to *MRE11^+/−^* cells. We also measured genomic dFdC incorporation in this mutant and found that the dFdC/hdC ratio (Fig. 6B) increased approximately fourfold in *MRE11^H129N/−^* cells compared to *MRE11^+/−^* cells.

To assess whether the Mre11 3′ to 5′ exonuclease activity, rather than the single strand endonuclease activity, contributes to dFdC resistance and removal, we tested dFdC sensitivity of both DT40 *MRE11^+/−^* and *MRE11^H129N/−^* cells treated with different concentrations of mirin, which has been shown to specifically inhibit the Mre11 3′ to 5′ exonuclease but not the endonuclease activity (*6*, *40*). Increasing concentrations of mirin (Fig. 6A, green and red lines) sensitized *MRE11^+/−^* cells to dFdC, whereas *MRE11^H129N/−^* cells were not sensitized beyond the sensitivity caused by the *MRE11^H129N/−^* mutation. We obtained similar results using another Mre11 exonuclease inhibitor, PFM39 (see fig. S4). After mirin treatment, the dFdC/hdC ratio increased approximately fourfold in *MRE11^+/−^* cells compared to untreated cells, while mirin treatment did not further increase the dFdC/hdC ratio in *MRE11^H129N/−^* cells (Fig. 6B). The observation that mirin treatment does not increase dFdC sensitivity or the dFdC/hdC ratio in *MRE11^H129N/−^* cells confirms that mirin specifically targets Mre11 (exo)nuclease activity and that the effects of mirin on dFdC sensitivity and the dFdC/hdC ratio in *MRE11^+/−^* cells are not due to interference of mirin with other (nuclease) activities.

We also measured the amount of constituent dC in genomic DNA for a subset of experiments which formed the basis for Fig. 6B. As shown in fig. S5, which depicts the number of genomic dFdC and hdC molecules per 10^4^ dC molecules in genomic DNA, the increased dFdC/hdC ratio in mirin-treated *MRE11^+/−^* cells and in *MRE11^H129N/−^* cells results from an increase in dFdC accompanied by a smaller increase in hdC. This suggests that the Mre11 nuclease activity removes (chain-terminating) dFdC together with a stretch of newly synthesized DNA.

We also tested the effect of mirin on dFdC sensitivity and the dFdC/hdC ratio in human lung fibroblast MRC-5 cells. As shown in Fig. 6C, mirin treatment increased the sensitivity of MRC-5 cells to dFdC (Fig. 6C, left) and also increased the dFdC/hdC ratio (Fig. 6C, right). Last, to assess whether the Mre11 endonuclease activity contributes to dFdC resistance, we treated MRC-5 cells with 60 μM Mre11 endonuclease inhibitor PFM03 (*6*) and found that it does not increase dFdC sensitivity (Fig. 6D). Our observations suggest that the Mre11 3′ to 5′ exonuclease activity, but not the endonuclease activity, contributes to dFdC resistance and removal in DT40 and MRC-5 cells.

## DISCUSSION

Treatment with DNA damaging and replication inhibiting agents is one of the mainstays of cancer therapy. While defects in DNA repair pathways associated with carcinogenesis can sensitize cancer cells to treatment, it remains largely unknown which (often redundant) repair pathways/activities are responsible for repairing DNA damage caused by commonly used cancer drugs. Identification of and mechanistic insight into these pathways is important to improve treatment efficacy by tailoring the choice of drug to DNA repair defects present in cancer cells (personalized medicine) or by exploiting synthetic lethality with novel DNA repair–inhibiting drugs (*45*).

Artificial chain-terminating nucleoside analogs (e.g., dFdC and cytarabine) are extensively used not only in cancer treatment but also in antiviral therapy (*46*). Whereas nucleoside analogs used in cancer treatment target the replication machinery in the cancer cell, antiviral nucleoside analogs are designed to inhibit viral replication; integration into the genomic DNA of the host cell causes unwanted side effects (*47*). Therefore, identification of cellular activities that resist treatment with these drugs is also important to understand the off-target effects of (novel) antiviral nucleoside analogs.

While recent studies in yeast and human cells have implied a role for the Mre11 3′ to 5′ exonuclease activity in DSB end resection in meiotic and nonmeiotic cells, its role during replication has remained elusive. The 3′ to 5′ exonuclease activity of recombinant yeast Mre11 acts on blunt-ended double-strand DNA or a 3′ DNA end with a 5′ overhang but is not active toward a 3′ overhang (*19*). This activity is thus ideally suited to remove nucleotide analogs from the nascent 3′ end during replication. In this study, we have shown that inhibition of the Mre11 3′ to 5′ exonuclease activity sensitizes cells to dFdC and leads to an increase in dFdC in genomic DNA. This suggests that Mre11 exonuclease activity removes dFdC from genomic DNA during DNA replication, contributing to the innate resistance of cells against this drug.

Most previous studies on replication fork stalling have concentrated on stalling caused by nucleotide pool depletion or obstacles on the template strand (*20*). Under treatment conditions used in our study to quantify the dFdC/hdC ratio in genomic DNA, cells arrest at the first opportunity for dFdC to be integrated into genomic DNA, which is S phase. To the best of our knowledge, our study is the first to identify an activity that removes a chain-terminating nucleoside analog from the nascent (rather than the template) strand during DNA replication in vivo.

Our study demonstrates a role for Mre11 nuclease activity in dFdC removal in the model eukaryote *S. pombe*, in chicken DT40 cells, and in human MRC-5 cells, suggesting that this role in removing chain-terminating nucleotides from DNA has been conserved during evolution. While cells are unlikely to encounter dFdC in the natural environment, some recent studies suggest that chain-terminating nucleosides are present within the cellular free nucleoside/nucleotide pool, which is prone to endogenous damaging modifications; free nucleotides are much more susceptible to modifications than nucleotides already incorporated into DNA (*48*, *49*). While the effect of integration of naturally occurring noncanonical nucleotides into genomic DNA is an underresearched area of study, there is evidence from biochemical assays with human proteins that incorporation of the oxidized nucleotide 8-oxo-dGTP into genomic DNA delays or terminates chain elongation (*50*, *51*). It is, thus, possible that the Mre11 nuclease activity has evolved to remove noncanonical, endogenously damaged chain-terminating nucleotides from DNA during replication.

Previous studies have shown involvement of MRE11 exonuclease activity in uncontrolled deleterious degradation of DNA at the replication fork in cells that are deficient for HR (e.g., when RAD51 or BRCA2 are mutated/depleted); however, these studies have not revealed a beneficial role of Mre11 exonuclease activity in supporting progression of stalled forks in recombination-proficient cells. Our observations that the Mre11 exonuclease activity preferentially removes dFdC over hdC (which is chemically indistinguishable from dC) and that deletion of *exo1* does not lead to an increase in the dFdC/hdC ratio show that the Mre11-dependent dFdC removal activity we uncovered is not the result of indiscriminate resection and is, thus, distinct from the previously described role of Mre11 nuclease activity in the uncontrolled deleterious resection resulting from defective HR.

We have thus uncovered a previously unidentified role for the Mre11 exonuclease activity in supporting replication fork progression, which has major implications for our understanding of the evolutionary conserved role of Mre11 in unblocking stalled replication forks. These findings will form a basis for future studies into the role of Mre11 and other (nuclease) activities in the removal of naturally occurring endogenously damaged nucleosides and artificial nucleoside analogs used in cancer and antiviral therapy.

## MATERIALS AND METHODS

### *S. pombe* strains and techniques

For strain construction and propagation, standard genetic methods and media were used (*52*). Strains used and constructed in this study are listed in table S1. For spot tests, cultures were diluted to 10^7^ cells/ml and 10-fold diluted to 10^2^ cells/ml. Of these dilutions, 10 μl were spotted for each culture on each plate.

The *dCK* gene (*34*) under control of the *S. pombe adh* promoter, was integrated into the *S. pombe* genome, replacing *ura4*. Subsequently, the *hENT1* gene (*33*), under control of the *adh* promoter, coupled to a nourseothricin resistance marker, was integrated adjacent to dCK. Strains containing *dCK* and *hENT1* were grown on minimal media (Edinburgh Minimal Medium and glutamate), as they display slow growth and elongated cell phenotype on yeast extract. For the high-performance liquid chromatography (HPLC)–based quantification of free cellular dNTP and dFdCTP pools, the procedure described in (*53*) was followed.

To assess replication progression using flow cytometry, asynchronous *nda3-KM311* cells were arrested in G_2_ by a 6-hour incubation at restrictive temperature (16°C) and released at permissive temperature (30°C) in the presence of dFdC. Samples were taken every 30 min and were processed for flow cytometry according to the standard procedures (*54*). 2D gel electrophoresis (in the absence of trimethylpsoralen) was performed, as described (*42*), using the AseI/BamHI ura4^+^ fragment.

For mass spectrometric detection of genomic dFdC and hdC, a 100-ml *S. pombe* culture at a density of 4 × 10^6^ cells/ml was treated for 3 hours with 50 μM dFdC and 50 nM hdC. Cells were harvested and genomic DNA was isolated, as previously described (*55*), with minor modifications (detailed protocol available on request).

### DT40 and MRC-5 cell lines and techniques

DT40 (*56*) and MRC-5 (SV40 transformed; provided by A. R. Lehmann, University of Sussex) cells were maintained using standard methods and media. The DT40 *MRE11^H129N/−^* nuclease mutant was created from *MRE11^+/−^* cells (*57*) provided by S. Takeda (Faculty of Medicine, Kyoto University) by a targeted knock-in, leaving *MRE11* under control of its own promoter, and apart from the H129N mutation in exon 4 and integration of a puromycin resistance marker in the intron downstream of this mutation, leaving the overall intron/exon structure intact.

For DT40 colony survival assays, cells were exposed to drugs for 24 hours in liquid medium, before being plated in methylcellulose-containing media (*56*). For MRC-5 colony survival assays, cells were exposed to drugs for 24 hours before being processed (*58*).

The MTS assay was carried out using a CellTiter 96 AQueous One Solution Cell Proliferation Assay kit (Promega). DT40 cells (50,000 per well) were incubated for 4 hours at 39°C, after which drugs and compounds were applied. Cells were incubated for an additional 48 hours at 39°C. MTS reagent (20 μl) was added to each well, and cells were incubated for 4 hours.

Absorbance was read at 490 nm using a microplate reader and corrected for the absorbance of wells containing medium without cells or drugs. Rates of metabolic activity were expressed as a percentage relative to untreated control cells.

For mass spectrometric detection of genomic dFdC and ^13^C^15^NdC, 5 ml of DT40 (1 × 10^6^ cells/ml) were treated with 500 nM dFdC and 10 nM ^13^C^15^NdC and varying doses of Mirin for 24 hours. MRC-5 cells were grown to 50% confluence in a 100-mm cell culture dish and treated with 10 nM ^13^C^15^NdC and 1 μM dFdC for 24 hours. Genomic DNA was isolated using the QIAGEN DNeasy Blood and Tissue Kit.

### Preparation of genomic DNA and nucleoside quantification by LC-MS/MS

Genomic DNA obtained from *S. pombe*, DT40, or MRC-5 cells was hydrolyzed and dephosphorylated by a combination of nuclease P1 (Sigma-Aldrich, N8630), phosphodiesterase I (Sigma-Aldrich, P3243), and alkaline phosphatase (Sigma-Aldrich, P5931) into single nucleosides, as previously described (*59*). Nucleosides were separated using a C18 reverse-phase HPLC column and quantified by an in-line triple quadrupole mass spectrometer. The mass/charge ratio transitions used for detecting the various nucleosides were as follows: dFdC, 264.1 → 112.1; hdC, 231.1 → 115.0; ^13^C^15^NdC, 240.1 → 119.1; dC, 228.1 → 112.1; deoxyadenosine, 252.1 → 136.1; deoxyguanosine, 268.1 → 152.1; and thymidine, 243.1 → 127.1. A detailed protocol is available on request. Using this methodology, we typically detect ~1 dFdC per 16,000 dC in WT DT40 cells not treated with mirin; this is well above the dFdC detection limit, which we estimate to be ~1 dFdC per 70,000 dC.

### Statistics

Statistical significance was tested using the (paired or unpaired) Student’s *t* test. *P* < 0.05 was considered to be significant. Analysis was based on at least three independent experiments, for details see Results and figure legends.

## Supplementary Material

aaz4126_SM.pdf

## SUPPLEMENTARY MATERIALS

Supplementary material for this article is available at http://advances.sciencemag.org/cgi/content/full/6/22/eaaz4126/DC1

## Appendix

View/request a protocol for this paper from *Bio-protocol*.
