# Supplementary material for: Mre11 exonuclease activity removes the chain-terminating nucleoside analog gemcitabine from the nascent strand during DNA replication
Source: Sci Adv. 2020 May 29;6(22):eaaz4126. doi: 10.1126/sciadv.aaz4126 (PMC7259961; doi:10.1126/sciadv.aaz4126)
Supplement: aaz4126_SM.pdf [file aaz4126_SM.pdf]

Science Advances
AAAS

advances.sciencemag.org/cgi/content/full/6/22/eaaz4126/DC1

Supplementary Materials for

Mre11 exonuclease activity removes the chain-terminating nucleoside analog gemcitabine from the nascent strand during DNA replication

L. Boeckemeier, R. Kraehenbuehl, A. Keszthelyi, M. U. Gasasira, E. G. Vernon, R. Beardmore, C. B. Vågbø, D. Chaplin, S. Gollins, H. E. Krokan, S. A. E. Lambert, B. Paizs, E. Hartsuiker*

*Corresponding author. Email: e.hartsuiker@bangor.ac.uk

Published 29 May 2020, *Sci. Adv.* **6**, eaaz4126 (2020)
DOI: 10.1126/sciadv.aaz4126

This PDF file includes:

Table S1
Figs. S1 to S5

Table S1. *S. pombe* strains used in this study

Strain	Genotype
EH722	*h*$^+$
MG71	*h*$^+$ *ura4::nat-hENT1*
MG85	*h*$^+$ *ura4::dCK-nat-hENT1 ura4-aim*
MG70	*h*$^+$ *ura4::dCK-nat-hENT1*
RO602	*h*$^+$ *ura4::dCK-nat-hENT1 ura4-aim*
AK290	*h*$^+$ *ura4::dCK-nat-hENT1 mre11-D65N ura4-aim*
MG119	*h*$^+$ *ura4::dCK-nat-hENT1 rad50::kan ura4-aim*
AK278	*h*$^+$ *ura4::hsdCK-nat-hENT1 mre11::kan ura4-aim*
MG296	*h*$^+$ *ura4::dCK-nat-hENT1 nbs1::kan ura4-aim*
AK274	*h*$^+$ *ura4::hsdCK-nat-hENT1 ctp1::kan ura4-aim*
EH1118	*h*$^-$ *smt0 ura4::dCK-nat-hENT1 mre11-D65N*
EH1119	*h*$^-$ *smt0 ura4::dCK-nat-hENT1*
EH1308	*h*$^-$ *smt0 ura4::dCK-nat-hENT1 loxP-mre11*$^+$*-loxM*
EH1290	*h*$^-$ *smt0 ura4::dCK-nat-hENT1 loxP-mre11-H134S-loxM*
EH1306	*h*$^-$ *smt0 ura4::dCK-nat-hENT1 loxP-mre11-H68S-loxM*
RO653	*h*$^-$ *smt0 arg7*$^+$*::dCK his7*$^+$*::hENT-nat nda3-KM311*
RO666	*h*$^-$ *smt0 arg7*$^+$*::dCK his7*$^+$*::hENT-nat nda3-KM311 mre11-D65N*
EH01152	*h*$^+$ *arg7*$^+$*::dCK his7*$^+$*::hENT-nat*
EH1171	*h*$^+$ *loxP-mre11*$^+$*-loxM ura4::adh-hsdCK*
EH1168	*h*$^+$ *mre11-H134S, ura4::adh-hsdCK*
EH1172	*h*$^+$ *mre11-H68S, ura4::adh-hsdCK*
AK312	*h*$^+$ *mre11::kan ura4-aim*
MG273	*h*$^+$ *ura4::dCK-nat-hENT1 exo1::ura4*

Figure S1

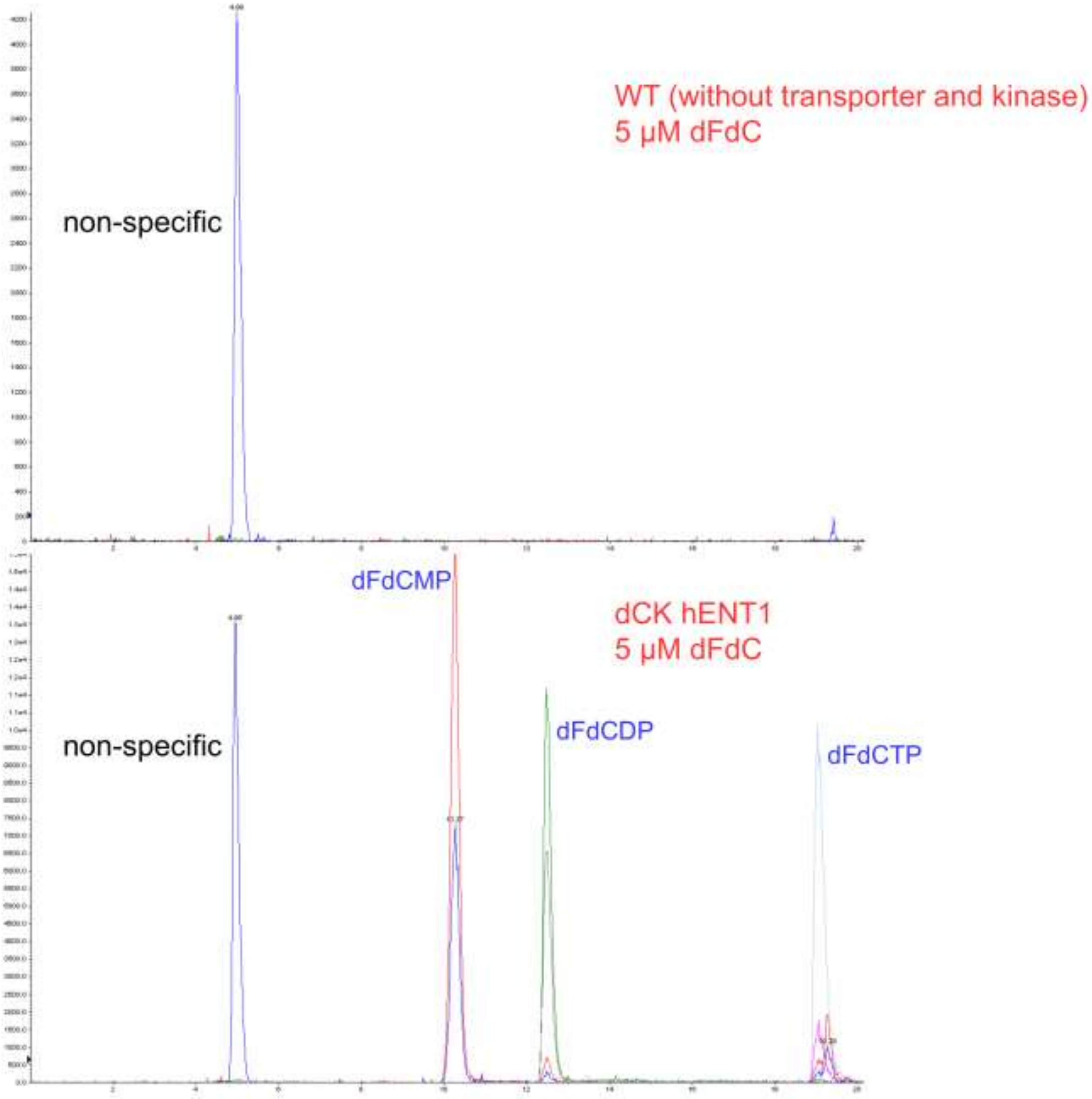

Mono-, di- and tri-phosphorylated dFdC metabolites are present in cells expressing *hENT1* and *dCK*
Extracted ion chromatogram for $[M+H]^+$ ions of dFdCMP, dFdCDP and dFdCTP in positive ion mode. *S. pombe* cells bearing the human equilibrative nucleoside transporter 1 (hENT1) and human deoxycytidine kinase (dCK) (lower panel), and without transporter and kinase (upper panel), were treated with 5 µM dFdC for 3 hours, nucleotides were extracted as described (Kumar et al., 2010). Intracellular phosphorylation of dFdC was measured by LC-MS/MS. Mass-to-charge (*m/z*) transitions: dFdCMP: 344.0 to 112.1 (red), 344.0 to 246.1 (dark blue); dFdCDP: 424.0 to 326.0 (green), 424.0 to 112.1 (grey); dFdCTP: 504.0 to 326.0 (light blue), 504.0 to 112.1 (purple). *m/z* transitions characteristic for dFdCMP can also be observed at the elution times for dFdCDP and dFdCTP, presumably due to in-source fragmentation. The identity of the non-specific signal (*m/z* transition 344.0 to 246.1, dark blue) at retention time 4.98 min. in the control sample and at 4.95 min. in the dFdC treated sample, is unknown.

Figure S2

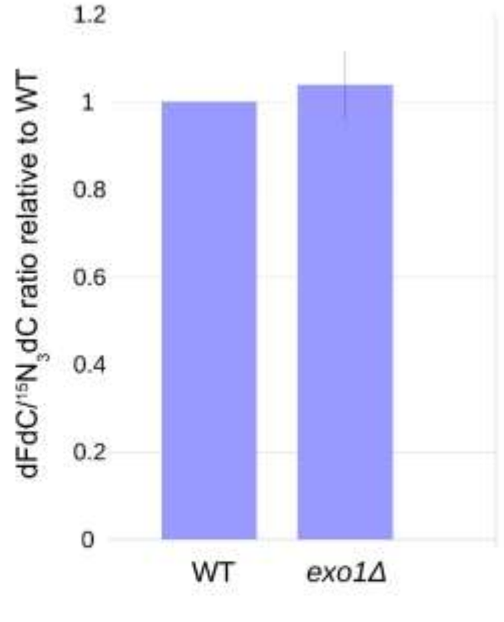

An *exo1* deletion mutant does not show an increase in the dFdC/hdC ratio
A strain deleted for *exo1* does not show an increase in the dFdC/hdC ratio compared to WT. Error bar depicts standard error, *n*=3.

Figure S3

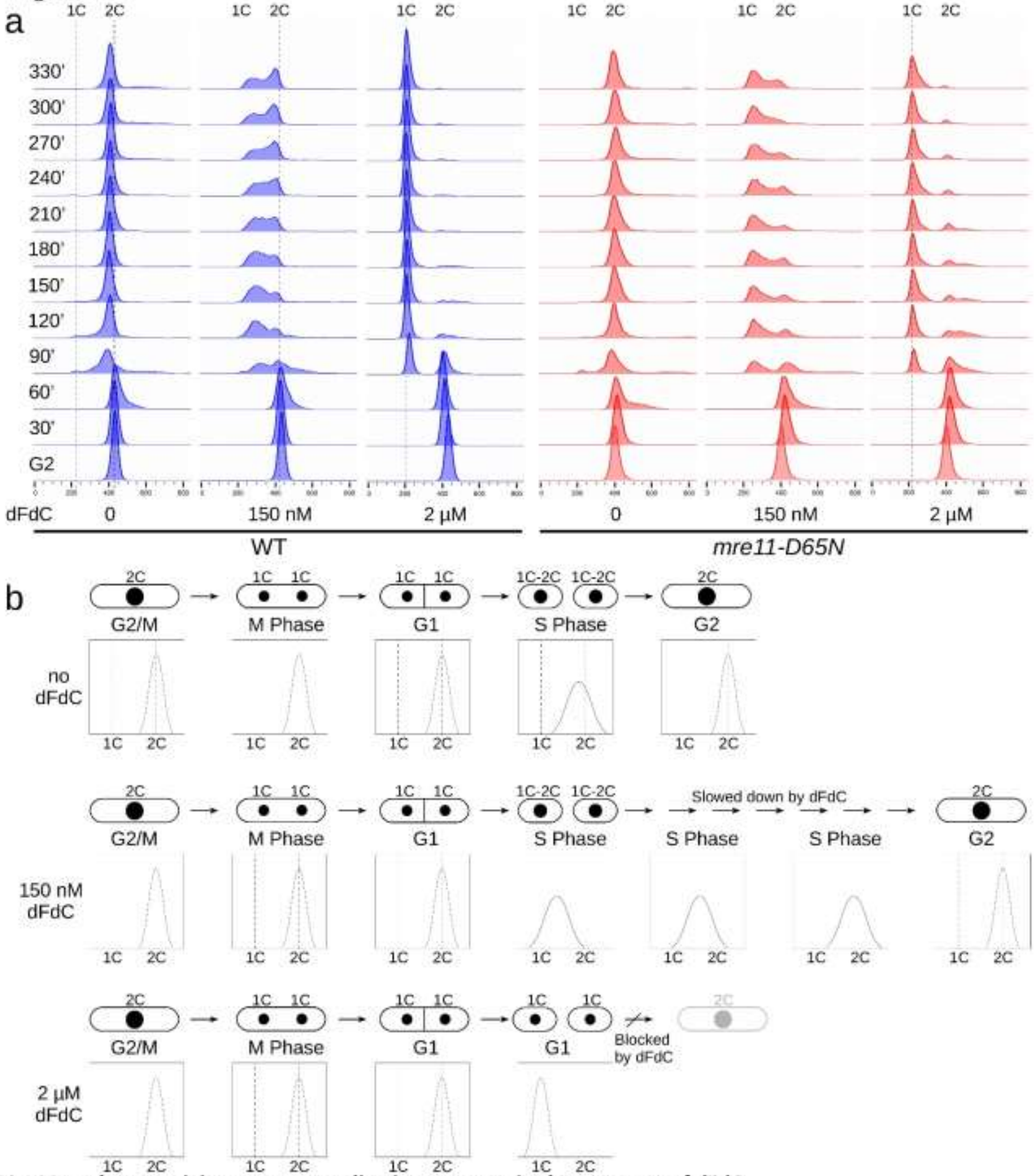

Mre11 nuclease activity supports replication progress in the presence of dFdC

a) Alternative presentation of data shown in Fig. 4a. FACS analysis of WT (blue) and *mre11-D65N* (red) cells, synchronised and released in G2 in the presence of 0, 150 nM and 2 µM dFdC. As *S. pombe* cells have a very short G1 phase and will initiate DNA replication directly after G1, without dFdC, the shift from G2 to G1 is barely visible for both WT and *mre11-D65N* cells (see diagram in panel b). In contrast, when treated with 2 µM dFdC, both WT and mutant cells display predominantly a 1C DNA content after mitosis and progression to 2C is blocked. In the presence of 150 nM dFdC, WT cells slowly progress through S-phase from 1C to 2C, whereas *mre11-D65N* cells mainly display a 1C DNA content throughout the time course. **b)** Diagram showing interpretation of WT FACS profiles shown in panel a. *nda3* cells are arrested at G2/M at restrictive temperature. After release from this arrest in the absence of dFdC, cells will quickly go through mitosis. Before cell division takes place, the two G1 (1C) nuclei remain together, separated by a septum; these cells will show a DNA content of 2C (or higher). DNA replication is initiated around the time of septation but before cell division, so that cells never show a 1C DNA content. In the presence of 150nM dFdC, DNA replication is slowed down, so that the DNA content at the time of cell division is lower than in untreated cells (but higher than 1C); the DNA content of the cells slowly progresses towards 2C. Finally, in the presence of 2 µM dFdC, replication is blocked and after cell division cells display a 1C DNA content.

Figure S4

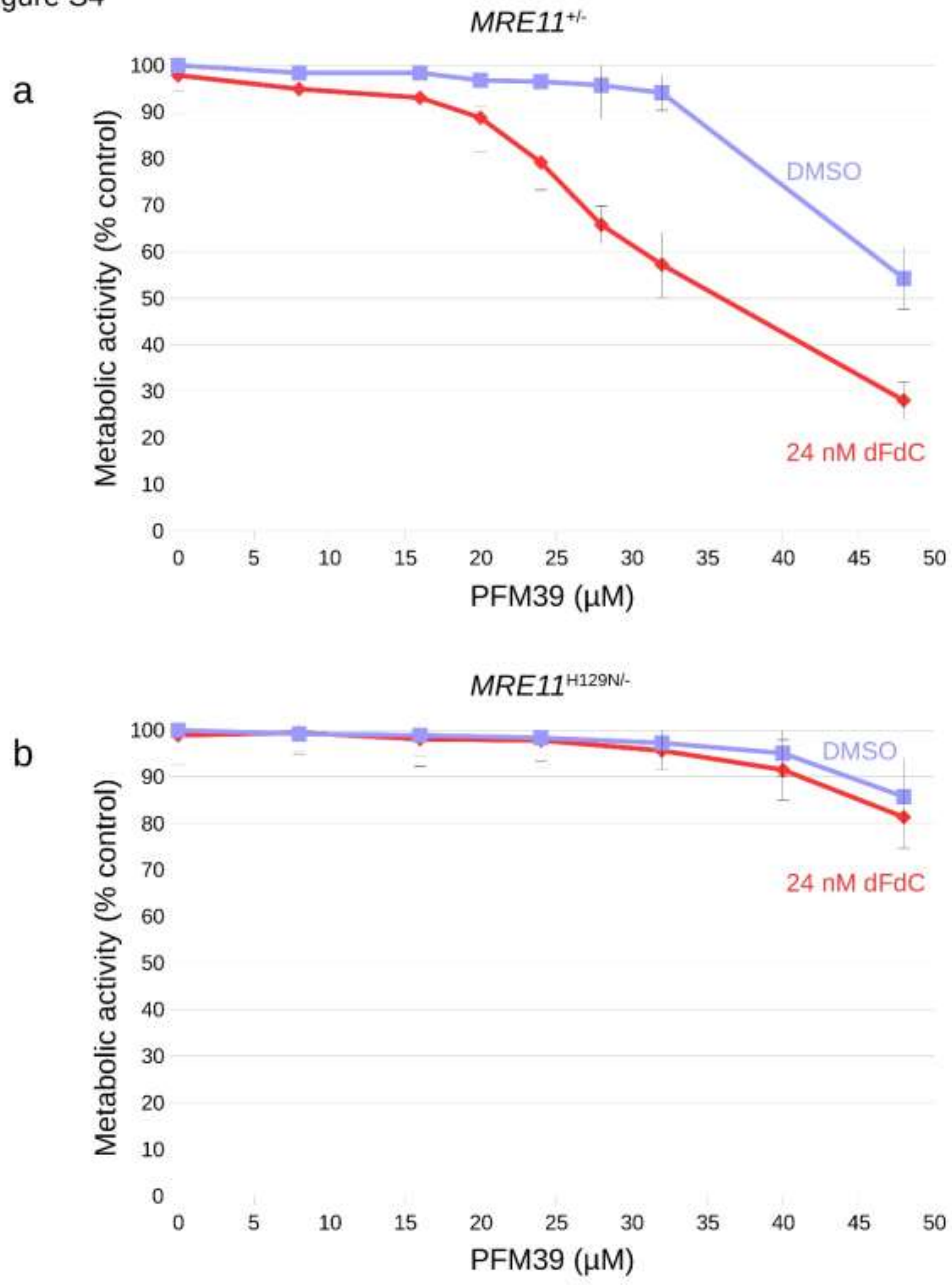

The Mre11 exonuclease inhibitor PFM39 sensitises *MRE11*$^{+/-}$ cells to dFdC

Cells where treated with increasing concentrations of PFM39 in the absence or in the presence of 24 nM dFdC. Metabolic activity, as determined by the MTS assay, is relative to the metabolic activity of DMSO treated cells in the absence of PFM39 (100%).

a) Increasing PFM39 concentrations sensitise *MRE11*$^{+/-}$ cells to dFdC. **b)** Nuclease dead *MRE11*$^{H129N/-}$ cells treated with increasing PFM39 concentrations are not sensitised to dFdC, suggesting that PFM39 specifically targets the Mre11 nuclease activity.

Figure S5

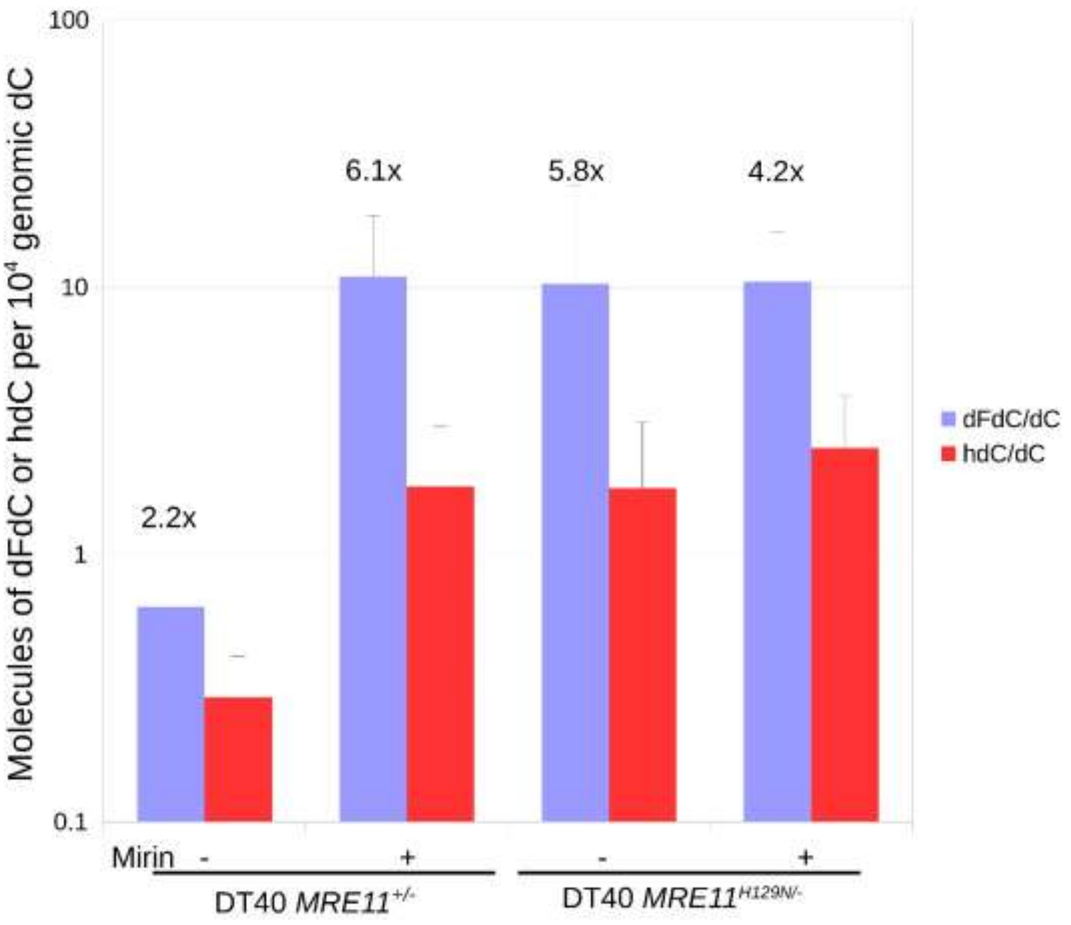

Molecules of dFdC or hdC per 10^4 constituent dC in genomic DNA of DT40 cells.
Treating *MRE11*$^{+/-}$ cells with 500 nM dFdC and 10 nM hdC (as described in Materials and Methods) leads to an integration rate of 0.6 dFdC per 10^4 dC. After Mirin treatment and in the *MRE11*$^{H129N/-}$ mutant, the dFdC rate is increased to ~10 dFdC per 10^4 dC. In *MRE11*$^{+/-}$ cells we detect 0.3 hdC per 10^4 dC, after Mirin treatment and in the *MRE11*$^{H129N/-}$ mutant this is increased to ~2 hdC per 10^4 dC. The dFdC/hdC ratio is indicated above the bars. Data are from 3 of the 7 experiments presented in Fig. 6. Error bars depict standard error, n=3.
